# Toward a Working Definition of eCohort Studies in Health Research: Narrative Literature Review

**DOI:** 10.2196/24588

**Published:** 2021-01-21

**Authors:** Vasileios Nittas, Milo Alan Puhan, Viktor von Wyl

**Affiliations:** 1 Epidemiology, Biostatistics and Prevention Institute University of Zurich Zurich Switzerland

**Keywords:** cohorts, digital epidemiology, eCohorts, eHealth

## Abstract

**Background:**

The wide availability of internet-connected devices and new sensor technologies increasingly infuse longitudinal observational study designs and cohort studies. Simultaneously, the costly and time-consuming nature of traditional cohorts has given rise to alternative, technology-driven designs such as eCohorts, which remain inadequately described in the scientific literature.

**Objective:**

The aim of this study was to outline and discuss what may constitute an eCohort, as well as to formulate a first working definition for health researchers based on a review of the relevant literature.

**Methods:**

A two-staged review and synthesis process was performed comparing 10 traditional cohorts and 10 eCohorts across the six core steps in the life cycle of cohort designs.

**Results:**

eCohorts are a novel type of technology-driven cohort study that are not physically linked to a clinical setting, follow more relaxed and not necessarily random sampling procedures, are primarily based on self-reported and digitally collected data, and systematically aim to leverage the internet and digitalization to achieve flexibility, interactivity, patient-centeredness, and scalability. This approach comes with some hurdles such as data quality, generalizability, and privacy concerns.

**Conclusions:**

eCohorts have similarities to their traditional counterparts; however, they are sufficiently distinct to be treated as a separate type of cohort design. The novelty of eCohorts is associated with a range of strengths and weaknesses that require further exploration.

## Introduction

### Background

The term “cohort” is derived from Latin and was initially used to describe Roman military units; its epidemiological meaning describes a defined group of people, observed over a period of time to determine certain health outcomes [1,2]. Cohort studies provide invaluable information on the determinants of health, disease, and death [1]. Much of modern medicine’s knowledge, including the consequences of smoking and alcohol, the impact of socioeconomic factors on health outcomes, and the role of physical activity on chronic disease, is the result of large cohort studies [3-5]. Nonetheless, performing these studies remains a largely complex, expensive, and time-consuming endeavor, often embedded within resource-limited environments [6]. These limitations have led to the development of novel technology-driven approaches that aim to mitigate some of these challenges [7,8].

eCohorts, also often referred to as online or web-based cohorts, are the inevitable result of recent technological advances as well as societal developments. eCohorts harness the reach and flexibility of the internet to deal with some of the inherent complexities of traditional approaches, including the need for time-consuming and costly recruitment of large sample sizes, slow communication methods, and participant retention [8,9]. The growing acceptance of mobile health and wearables has enabled the continuous and relatively simple self-monitoring of health and risk, where patients can generate and access their personal health data supported by health apps that provide personalized content, ranging from primary prevention to therapy support and rehabilitative coaching [10-12]. In parallel, as landline telephone and postal communication use declines, social media and online communities are increasingly being used as platforms for health information sharing, in which users actively engage and contribute [8,9]. eCohorts are shaped by these developments, which promise reach, flexibility, retention, and efficiency [8,9].

### Defining “eCohort”

A clear definition of what constitutes an eCohort study has not yet been established. Existing research uses a variety of terms, including “web-based,” “online,” “digital,” and “internet” cohorts, while emphasizing different methodological aspects from web-based recruitment to online data collection and digital follow up [7,8,13,14]. We believe that the first step toward establishing a comprehensive eCohort definition is to perform a methodological comparison of traditional cohorts to eCohorts, considering all steps in the design and life cycle of these studies. The main research questions addressed were as follows: (1) Can we define eCohorts based on what we know about traditional cohorts? (2) How similar or how different are eCohorts from traditional cohorts? Thus, the primary aim of this study was to provide the first directions toward answering these questions.

### Aims

Based on a literature search and our own experiences, we aimed to outline and discuss what may constitute an eCohort and how these elements can be brought together to formulate a first working definition for health researchers. This definition should go beyond basic technical characteristics to provide a holistic description of all steps along the life cycle of an eCohort study, as well as its distinct strengths, weaknesses, risks, and challenges. As a first step to achieve this goal, we conducted a literature search to contrast the characteristics of eCohorts with those of well-defined traditional cohorts, which can facilitate a better understanding of their differences and potential similarities. We also aim to use the findings of this narrative review to inform the design of an upcoming comprehensive scoping review on eCohorts.

## Methods

**Figure 1:**
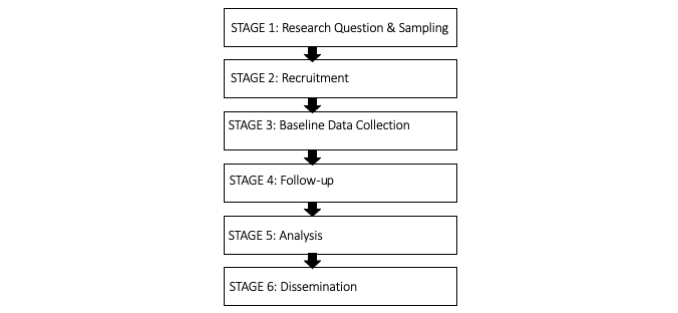
Core stages in the life cycle of a cohort study.

Our approach was based on a two-staged iterative review and synthesis process. The paper is organized as follows. Initially, we compare traditional and eCohort studies across the 6 core steps in their life cycle as outlined in Figure 1. We then continue discussing eCohorts in the context of additional characteristics such as flexibility, interactivity, usability, security, scalability, and costs. Our synthesis relied on a (1) narrative literature synthesis and (2) our own experiences with traditional cohorts and eCohorts, such as with the Women’s Interagency HIV Study [15], Swiss HIV Cohort Study [16], and Swiss Multiple Sclerosis Registry [17].

We searched PubMed and Google Scholar (first 5 pages) using the terms “cohort profile,” “cohort description,” “cohort methods,” as well as “e-cohort,” “web-based cohort,” “online cohort,” and “digital cohort.” For traditional cohorts, to avoid an unmanageable number of hits, we set the filter to observational studies in PubMed, which automatically captures only publications from 2012 onward. To include older cohorts, we used Google Scholar. This was followed by the selection of 10 traditional cohort and 10 eCohort studies. As we did not aim to provide a detailed synthesis of all existing literature, we arbitrarily set the cutoff at 10, based on narrowing down iteratively and pragmatically. Our selection was guided by the criteria outlined in Textbox 1. First, we selected studies with titles and abstracts that clearly indicated a detailed methodological account. We then proceeded iteratively to select 10 traditional and 10 eCohort studies that together provided the most rich and broad methodological information, with as few as possible overlaps, while also fulfilling the third criterion of Textbox 1.

**Literature review inclusion criteria.**

- Criterion 1: each publication should provide sufficient descriptive information on at least three of the stages in the life cycle of a cohort study (see Figure 1).
- Criterion 2: in total, the set of included publications should provide sufficient content on all stages in the life cycle of a cohort study.
- Criterion 3: in total, the set of included publications should provide a good balance between older and newer cohorts as well as between general population and disease-specific cohorts.

The seven life-cycle stages of a cohort study guided our data extraction procedure (Figure 1). Each stage received a code and for each code we iteratively developed several subcodes. The subcodes emerged during the full-text appraisal. Coded sections were then transferred to an Excel file, and were synthesized and analyzed thematically. Multimedia Appendix 1 provides a list of our codes.

## Results

### Included Studies

PubMed yielded 275 hits for traditional cohorts and 46 hits for eCohorts. Google Scholar yielded an additional 150 publications for traditional cohorts and 200 publications for eCohorts. Following our inclusion criteria, we selected 10 illustrative traditional and 10 eCohort publications, which are listed in Table 1.

**Table 1:** Included cohort studies.

Cohort name		Year of start	Reference
**Traditional cohorts**			
	Framingham Heart Study	1948	Tsao and Vasan [18]
	The National Child Development Study	1958	Power and Elliott [19]
	Nurses’ Health Study	1976	Bao et al [20]
	Swiss HIV Cohort Study	1988	Schoeni-Affolter et al [16]
	Cohort of Norway (CONOR)	1994	Næss et al [21]
	The Danish National Birth Cohort	1996	Olsen et al [22]
	PIAMA Birth Cohort	1996	Wijga et al [23]
	UK Millennium Cohort Study	2000	Connelly and Platt [24]
	The Chronic Kidney Disease in Children Cohort Study	2005	Furth et al [25]
	The lidA Cohort Study	2011	Hasselhorn et al [26]
**eCohorts**			
	NINFEA Birth Cohort	2005	Firestone et al [8]
	The Nurses and Midwives e-cohort Study	2006	Turner et al [9], Huntington et al [27]
	Snart-Gravid Cohort	2007	Christensen et al [14]
	ELF Cohort	2007	Firestone et al [8]
	Etude NutriNet-Santé e-cohort	2009	Andreeva et al [13], Andreeva et al [28], Hercberg et al [29]
	UK Cosmos	2009	Toledano et al [30]
	SnartForaeldre	2011	Christensen et al [14]
	French G-GrippeNet cohort	2014	Loubet et al [31]
	Swiss MS Registry	2016	Puhan et al [7]

### Stage 1: Research Question and Sampling

Every well-grounded epidemiological study is based on a well-defined research question. Traditionally, cohort studies are based on broad and multipurpose questions, dynamically changing over time based on new insights, theory, and expected future challenges [16,19,21-23,26]. New questions are commonly answered by previously collected data, as these are usually rich and highly practical. eCohort research questions do not deviate substantially from this traditional approach. Nonetheless, eCohorts are more easily aligned with the principles of citizen science (eg, involving patients in the process) and may entail technology validation components (eg, exploring the use of technology in the implementation of cohort studies) [7,9,27]. Although eCohort questions may also dynamically change over time, they are usually answered prospectively rather than using existing data, enabled by lower logistical hurdles and higher flexibility.

Identifying and recruiting a sample of adequate size and representativeness is a crucial and yet challenging step of cohort studies. Traditional cohort designs rely on well-established sampling processes, aiming for samples that are representative of the target population in terms of characteristics that potentially impact results (eg, measures of disease occurrence or specific associations). Commonly, participants are randomly selected from a predefined population group (eg, random sample of all inhabitants in a city), defined by specific events (eg, births within a certain period), and framed around specific exposures or diseases, as well as combinations of these, which may include multiple stages and stratification schemes [6,18,20,21,23,24,26]. For eCohorts, if any actual sampling process takes place, it tends to be more inclusive and less systematic than that used for traditional cohort studies. Participants are usually self-selected volunteers who are reached through various online, as well as offline, community outreach and advertisement efforts [7,9,31].

### Stage 2: Recruitment

Study recruitment is the direct “engine” of any prospective cohort study. With an emphasis on sampling, potential participants are commonly preidentified and invited to participate, rendering comprehensive advertisement campaigns of lower importance. Traditional population-based cohorts primarily rely on traditional recruitment processes such as mailed invitation letters, and paper-based (or face-to-face) informed consent forms and reminders [18,20,21,23,25,26]. Participants are usually recruited and enrolled in a clinical context (eg, by physicians or nurses) [16,22,23,25]. Overall, the recruitment and study settings are very much interlinked with the clinical or community context.

By contrast, eCohorts are less attached to a clinical setting and instead rely on mixed, but mostly online and passive recruitment [9,13,27,31]. As samples are often self-selected, advertisement plays a key role. Beyond conventional methods (eg, flyers, posters), online advertising (eg, forums, social media) is becoming increasingly common, with invitations and reminders primarily sent digitally [8,9,14,27,29-31]. These approaches aim to direct potential study participants to dedicated web pages that provide all relevant study information and the option to register [8,14,29]. This is followed by the assignment of unique study identification codes and the completion of electronic consent if the legal context allows [7,9,13,27,30,31]. Self-selection, the unequal access to resources (eg, technology ownership), and the unequal distribution of skills (eg, digital literacy) may lead to selective samples of younger, better educated, high-income, health-conscious, and digitally affine participants, thereby impacting the external validity of eCohorts (ie, generalizability of study findings) [8,13,27,28,31].

### Stage 3: Baseline Data Collection

The collection of baseline information (eg, exposures, current health) sets the foundation of all future comparisons. At baseline, traditional cohorts usually rely on combinations of paper-based questionnaires, environmental surveys, existing records, medical examinations, biosampling, and interviews [6,16,18-20,22,23]. These approaches are now often complemented by web-based approaches (eg, online questionnaires), aiming to reduce printing and administrative costs [18]. In contrast, web-based data collection, mostly in the form of online surveys, is the norm in eCohorts [7,9]. Paper-based survey options and medical record data are used in a complementary manner to overcome limited digital literacy and validate self-reported information [7,9,30].

Traditional cohorts rely on multiple streams of data, which, if complete, are widely considered as valid and robust, allowing for multiple control mechanisms and information triangulation [21,25,26]. In contrast, the quality, reliability, and internal validity of digitally generated data, which constitute the core of eCohorts, remain under scrutiny. Information is primarily self-reported, and may be unstructured, incomplete, or generated by devices of unclear accuracy (eg, wearables). To mitigate these limitations, eCohorts often utilize customized, automated, interactive, and responsive online surveys that minimize missing or inaccurate data [8,14,27]. Skip logics remove irrelevant questions and improve user-friendliness, consistency checks and data entry formatting reduce missing data, intermitted saving options allow for questionnaire completion over multiple sittings, and altered and feedback messages ensure that inaccurate or incomplete information is kept to a minimum [7,14,27].

### Stage 4: Follow Up

Prospective cohort studies usually include longer follow-up periods, throughout which data are collected over multiple time points; this holds for both traditional and eCohort designs. Traditional designs use multiple approaches for follow up, which may be similar to the approaches used at baseline. These include regular mailed or telephone surveys, medical examinations, in-clinic biosampling, medical record linkages, data retrieval from disease and death registries, as well as personal interviews [18,19,21,22,26]. Attrition can be mitigated through record linkages (eg, school registries) that keep participant contact details updated, as well as by regularly requesting participants to update their records. Response rates are enhanced through repeated contact, combinations of multiple follow-up methods, as well as with the support of motivating health care professionals [19,20,24]. Although the time intervals between data collection points vary, they tend to be lengthy (eg, multiple years) [23,24,26].

The follow up of eCohorts is predominantly based on self-reported digital data collection (eg, online surveys, web-based diaries), which may or may not be complemented (or validated) by clinical data [7,29]. Although offline alternatives are not uncommon, they remain secondary [7]. Attrition is mitigated through (personalized) digital reminders (eg, email, SMS text messages, social media), as well as online requests to update contact details [9,27,30]. The flexibility of the internet equips eCohorts with a variety of tools to maintain response rates throughout the follow-up period. These include (1) participatory and citizen science approaches, (2) personalized and understandable feedback (eg, online data summaries), (3) tailored electronic reminders, and (4) interactive and responsive data collection methods (eg, real-time completion status, visual cues, error messages, instant feedback) [7,9,27,29-31]. Social networks (eg, Facebook, Twitter) may be utilized as contact and outreach tools, providing study updates, and allowing for direct and continuous contact with participants [7,30]. Biospecimen collection is rarer in eCohorts than in traditional cohorts, but can be accommodated remotely when needed, such as through mail-in self-kits [8]. Considering that most eCohort data are self-reported digitally, the time intervals between data collection points are flexible and more frequent than those used in traditional cohorts [31].

### Stage 5: Analysis

At the data analysis stage, concerns about data quality and approaches to mitigate these seem to be a key difference between traditional and eCohorts. The analyses of traditional cohorts are largely built upon combinations of pre-existing and prospectively collected clinical data (eg, medical records, biosampling results), which are widely considered as valid and robust [21,25,26]. Subjective and self-reported data (eg, surveys) are validated through and complemented by parallel streams of clinical information (eg, health insurance claims) [21,25,26]. Although challenges and biases (eg, low response rates, loss to follow up, limited data usefulness, low sample representativeness, social desirability bias) are not uncommon, concerns inherent to data quality are minimized through the use of well-established data collection instruments, a combination of data streams, and increasingly modernized data transfer and storage practice [16,26]. Analyses usually follow lengthy data collection processes.

As outlined above (Stage 3: Baseline Data Collection), the quality and reliability of eCohort data are often scrutinized, requiring considerate data management efforts and careful adjustments to data collection instruments to mitigate a negative impact on analyses. Part of these efforts is the complementary use of clinical data (eg, medical records) to increase validity, reliability, and overall quality [7,29]. Recent analytic advances in multiple imputations of missing data have the potential to mitigate these problems in both traditional and eCohorts. Despite these challenges, digital data collection has its advantages. Data access is improved, while data collection time frames can be shorter, thereby facilitating the completion of preliminary analyses without the need for lengthy gap periods [27].

### Stage 6: Dissemination

Details on the dissemination of cohort findings were scarce in the included traditional cohort publications. Dissemination seems to be focused on scientific publications, which, if added to the lengthy data collection and analysis completion periods, seems to have a rather delayed character. Nonetheless, traditional cohorts may have dedicated websites through which publications and key findings can be retrieved. A further element that could be described as integral to the dissemination strategy of traditional cohorts is the use of findings for the development and dissemination of clinical tools such as risk prediction scores [18,23,25].

By contrast, the dissemination of findings received greater emphasis in the included eCohort publications. The internet (eg, websites, newsletters, and social media) seems to be the primary tool for communicating updates and findings [7,9,27,30]. As mentioned in the previous section, the flexibility of digitalization may allow for faster data access and therefore more possibilities for preliminary analyses. In turn, this enables more frequent communication of findings and less lengthy gaps between updates [7,27]. Communication of updates, relevant news, and findings, including community outreach (eg, by webinars) and presentations to health care staff, participants, and patients, may be a part of overall strategies for maintaining participant motivation and mitigating attrition [7,30]. An important opportunity arising from eCohort (and digital health) research is that of reproducibility. Although science is undoubtedly facing a reproducibility crisis, the internet and its inherent possibilities for data availability and accessibility may eliminate replication barriers [32]. As data and technology availability increases, the practical challenges and costs of replicating research (eg, rerunning analyses) diminish. This is further facilitated by initiatives such as open science registries that aim for transparency and wide access to public research data [32]. If done correctly, the findings of eCohorts can facilitate reproducibility and open science, turning a crisis into a strength.

An overall summary comparing traditional cohorts to eCohorts is shown in Table 2.

**Table 2:** Comparison of eCohorts to traditional cohorts.

Characteristic	eCohort	Traditional cohort
Research question	Broad, multipurpose, interdisciplinary questions; questions may be rooted in citizen science and attached to methodological elements (eg, use of technology in epidemiological studies), change dynamically, and may be answered prospectively	Broad, multipurpose questions; questions change dynamically and are mostly answered with existing data
Sampling	Usually nonrandom sampling with self-selected volunteers	Random samples or clinic populations defined by event, exposure, or disease
Recruitment	Primarily online advertisement (eg, webpages, newsletters, forums, social media), but can be complemented by offline approaches (eg, flyers, posters) Recruitment usually online, through dedicated study webpages, possible at any place, any time Electronic consent procedures	Primarily offline advertisement (eg, flyers, posters, newspaper advertisements), but increasingly complemented by online approaches Recruitment usually within clinical (eg, by health care providers) or community setting and appointment-based Consent procedures usually face to face and paper-based
Baseline data collection	Primarily online and usually directly reported by participants (eg, web-based surveys). Sometimes complemented by offline data collection (eg, mailed surveys) and nonself-reported data (eg, medical record data)	Primarily offline (eg, paper-based questionnaires, data retrieval from existing records, personal interviews), and may be combined with medical examinations and biosampling
Follow up	Primarily online and usually directly reported by participants (eg, web-based surveys, personalized email, or SMS text message reminders) Rarely linked to medical care. Use of internet (eg, study website, social media, newsletters) for outreach and participant contact/engagement Data quality, reliability, and internal validity may be a concern Data quality tradeoffs due to self-reporting; need for simpler questions, better data management, and user-friendliness	Primarily offline (eg, paper-based questionnaires, data retrieval from existing records, personal interviews, medical examinations, and biosampling, mailed reminders) Usually linked to medical care; personal relationship (or at least personal interactions) between participant and study coordinators Strong focus on data quality, reliability, and internal validity
Analysis	Usually built on self-reported data Easier data access, preliminary analyses possible in shorter time frames Analyses tend to have a stronger participant (patient) focus	Built upon multiple data streams, and a combination of clinical and self-reported data Longer process, preliminary analyses more difficult in short time frames Analyses tend to have a stronger clinical/biomedical focus
Dissemination	In addition to publications, through a variety of online channels (eg, websites, social media) More frequent dissemination of findings Dissemination may be a part of an overall strategy to keep participants engaged Opportunities for reproducibility and open science	Primarily focused on scientific publications Subject to larger time gaps Dissemination of findings in form of clinical tools (eg, risk scores)

### Additional Considerations for eCohorts

#### Flexibility and Interactivity

The digitalized nature of eCohorts allows for a certain degree of flexibility and interactivity along all stages. Internet-based recruitment and participation are not bound to a certain physical location and allow for a larger geographic reach, even if (prospective) participants are on the move [27]. Electronic data collection can be designed to be personalized and interactive, such as online questionnaires that provide real-time feedback (eg, error messages, completion status), which can be completed over multiple sittings and quickly accessed from anywhere for long periods [8,9,29]. Similarly, automated and tailored electronic reminders and follow ups such as through email, SMS, or social media allow for cheaper, faster, more frequent, and interactive communication, thereby rapidly connecting and diverting participants to study websites (eg, through click-through links) [30]. Study websites can be interactively designed, aiming to engage participants and enhance compliance [27].

#### Usability

Inherently, the design and functioning of eCohorts require a certain level of participant engagement. Participants have to proactively access and engage with study websites, independently self-register, and repeatedly self-report their data, often without any physical interaction with project staff or health care providers. Inevitably, to motivate and sustain this engagement, the usability of involved technology is central. Some examples include barrier-free and tailored digital interfaces, simple online recruitment and registration processes, flexible and personalized data collection approaches, as well as functioning control and guidance systems [7,8,29].

#### Ethics and Security

Security plays an equally important role in motivating and sustaining participation. Ethical and privacy issues are inherent to the internet, which comes with certain vulnerabilities and risks related to various stages of a cohort design, including recruitment, advertising, and data collection [7,9]. Targeted advertising (eg, through social media platforms) requires the use of data that might be considered as private (eg, demographics, education) before a person is even aware of a study’s existence and long before they consent to participate [33,34]. Along similar lines, showing interest in an online advertised study (eg, by clicking on an advertisement) leaves an online trail that can be easily used by advertising companies for further profiling and targeted commercial advertising [33,34]. The tracking of our online behavior is inherent to the internet; nonetheless, this is challenging from an ethical and privacy perspective, especially in the context of sensitive health research. Further issues may arise from a certain loss of control over advertising, especially if that involves the sharing of advertisements by third parties and through various social media networks. Such uncontrolled spread might lead to losing sight of where a cohort is promoted, as well as of potential comments or questions that might have been posted across the internet [35]. Obtaining informed consent is an essential aspect of recruiting participants in a cohort. When conducted in a face-to-face manner, questions and concerns can be addressed interactively, which is lost if informed consent is obtained online and without individual contact. Filling this gap requires carefully designed online consent procedures that are transparent, understandable, and contain all elements of regular informed consent [36]. Finally, the internet makes it easier for sensitive data to be accessed without authorization, as well as hacked or replicated [33]. Although individual risk can be kept low if data are anonymized, some argue that the ease in which digital information is linked, shared, and merged renders all data potentially identifiable or traceable [37]. Therefore, adequate security features that keep risk at a minimum are inevitable [7]. Some of these features include robust password protections, high-standard information technology security, encrypted communication and data transfer, strict access controls, data deidentification, as well as the separation of personal information and unidentifiable data [7]. The emphasis on security also increases the responsibility that participants themselves have to carry, including adequate password protection, correct communication with study sites, and ensuring that devices and software are up to date.

#### Scalability and Costs

The internet adds a significant resource for fostering scalability and breadth [8]. Being predominantly online, eCohorts have the advantage of not being limited by physical location, having a larger sampling frame, and reaching populations who might have been otherwise difficult to reach [8,14]. Data can be collected over large geographic areas, even across borders, fostering collaborations while being managed from a single site [27]. Low-cost online recruitment and data collection techniques, facilitated by social media and their wide reach, may allow for longer recruitment and follow-up periods, thereby adding scale without prohibitively burdensome financial requirements [8,30]. Scalability is commonly associated with high costs and immense complexity, which is a major barrier of traditional cohort designs [27,30]. Nonetheless, the inherent flexibilities of eCohorts have the potential to keep costs substantially lower than those of their traditional counterparts [8,27]. Targeted online advertising can increase efficiencies, while online recruitment, invitations, and data collection can reduce labor, printing, and mailing costs [9,14,27,30]. These cost-efficiencies can nonetheless be rapidly offset. Large eCohorts require adequate resources (eg, call center, information technology personnel, digital experts, technical backups) and extensive error testing for solving arising problems as well as dealing with participant queries, all of which are costly [30]. Additional costs can also occur for the design of web platforms and data collection instruments, as well as for subsequent data security infrastructures, both of which are essential for data quality and misuse prevention, requiring maintenance throughout the full study duration [27,30].

## Discussion

### Principal Findings

Our comparison of traditional cohorts to eCohorts suggests a certain level of conceptual overlap. Assuming that a large proportion of eCohorts is run by people experienced with traditional cohorts, this is not a surprise. Although the stages, overall aims, and methodological basis are fairly similar, their realization differs between traditional cohorts and eCohorts across several aspects. Knowledge of traditional cohorts can be used to understand the methodological aims of eCohorts; however, the same knowledge cannot be used to derive implementation of the latter. Therefore, we consider the design of an eCohort to be a variant of its traditional counterpart.

The novelty and flexibility of eCohorts inherently bring some advantages over traditional cohort designs. The reach of the internet allows for wider, more flexible advertisement and recruitment that is not limited to a single physical setting, and may cover larger geographic regions as well as cross borders [8,14,27]. In combination with electronic data collection methods, this flexibility ultimately allows for easier scale up at potentially lower costs [8]. Digital data collection may also enable easier data availability and access (by researchers and participants), faster analyses, and more frequent dissemination of findings, all of which may foster the interest and engagement of participants [7,27]. The internet does not simply enable a wider reach, but if utilized correctly, also provides a targeted, personalized, engaging, and participant-centered process [7,8,29]. Online processes require some degree of interactivity and participant proactiveness, both of which are enhanced by digital communication methods such as personalized emails, SMS, and social media [8,9,29,30].

Inevitably, with novelty comes new risks and challenges. One of these is the generalizability of findings resulting from digitally collected data. eCohort samples often consist of volunteers that may not be representative of reference populations, posing potential external validity limitations [13], which may apply to both questions on measures of disease risks and occurrence (ie, descriptive epidemiology) and on associations (ie, inferential epidemiology). In contrast, population subgroups with lower digital literacy skills might be systematically left out, often being those that face additional sociodemographic disadvantages [38]. Concerns around privacy, security, and transparency require constant attention, especially in relation to data access, ownership, and sharing. Added to these issues, vaguely formulated and nontransparent privacy regulations create novel ethical challenges that cannot be ignored [39,40]. Finally, eCohorts require technological and analytical expertise that is carefully combined with traditional epidemiological skills and an overall motivation to keep up with the fast pace of technological innovation [40]. The promise of improved data collection and management, as well as cost-efficiency, can only be realized with carefully designed digital interfaces, effective participation incentives, and data quality assurances, which, if missing, can lead to observed moderate to low response rates and offset costs [8,9,14,27]. At the same time, given the inherent challenges in the management and analysis of eCohorts described above, some classical concepts of observational epidemiology may require adaptation to electronic contexts, including self-selection, limited potential for data management, mitigation of information biases, missing data, as well as the systematic integration and analysis of external electronic data (eg, secondary data from medical records).

### Hybrid Designs

To address our aim of gaining a better understanding of eCohorts, we contrasted their design to more traditional approaches. Nonetheless, cohort studies that combine both digital and traditional elements are an increasingly common phenomenon. As indicated in our results, traditional cohorts might be enhanced by digital components such as online recruitment and data collection, while largely eCohorts may also include complementary offline elements such as physical recruitment, conventional advertising methods (eg, flyers, posters), paper-based data collection, as well as the inclusion or collection of clinical data and biospecimens. In the future, and as technology advances, hybrid cohort designs will likely be inevitable. Digitalization may support traditional cohorts to stay up to date, reach younger populations, and deal with increased mobility, while increasing efficiency and reducing costs. In turn, eCohorts may benefit from traditional approaches for reaching nondigitally native populations and increasing the validity of their data.

### Working Definition

Based on our findings, a working definition of epidemiological eCohort studies could be formulated as follows. eCohorts are a novel type of cohort study, which (1) use the internet and technology as the primary delivery mode across most stages, from advertisement to recruitment, follow up, and dissemination; (2) are not entirely physically linked to a clinical setting; (3) follow more relaxed, not necessarily random, sampling procedures; (4) are primarily based on self-reported, digitally collected data, and usually have a strong patient focus; and (5) systematically aim to leverage the internet and digitalization to achieve scalability and efficiencies. We consider studies that have technology and the internet as their basis, but include hybrid elements (eg, on-site recruitment, paper-based data collection) within the scope of that definition.

### Limitations

As this is relatively novel territory, we aimed for a mix of methodological control and iterative exploration, for which our findings need to be viewed in light of the following limitations. Our sample did not aim to provide a comprehensive picture of the existing literature, but rather mainly a snapshot of existing work to provide a good basis for a comparison of traditional and eCohort designs. For this purpose, we kept our searches simple and pragmatic, and our final selection of included studies was iterative. For traditional cohorts, we decided to use a more specific search, adding methodological terms to reduce the sensitivity and number of hits, whereas for eCohorts, we chose a more sensitive sample as we expected fewer hits. Of note, an extended search would also include prominent examples of digital studies, which may not strictly follow principles of cohort studies but bear close resemblance (eg, the Apple Heart Study, Project Baseline). Furthermore, very recent (unpublished) or currently ongoing eCohorts that have not been captured by our search might well emphasize additional key aspects (eg, sensor measurements in combination with telehealth consultations and patient-reported data), for which we deem a follow up of our work necessary. We aimed to counteract the potential impact of our iterative selection approach by complementing our findings with the research team’s experience in traditional and eCohort studies. Our findings are primarily framed from an epidemiological perspective, which strongly impacted our focus and ultimately the definition we propose. Capturing and fully understanding all aspects of eCohorts would require further research, ideally exploring eCohorts through various angles, including an eHealth and ethical perspective. Such work would ultimately help us further refine the definition and conceptualization of eCohorts.

### Conclusion

This study provides a working definition of eCohorts, facilitating a better understanding of their implementation from an epidemiological and traditional cohort perspective. Our synthesis indicates that eCohorts may have many similarities to their traditional counterparts; however, eCohorts are sufficiently distinct to be treated as a separate type of cohort design. Sampling and recruitment are more flexible, the use of the internet and technology is prominent across all cohort stages, and analyses are primarily based on self-reported and digitally collected data. The novelty of eCohorts comes with a range of strengths, weaknesses, as well as uncertainties that require further exploration. Finally, eCohorts inherently offer new insights on how the internet and emerging technology can contribute to and blend in with epidemiological and broader health research.

## Appendix

Multimedia Appendix 1 Coding.
