# Supplementary material for: Toward a Working Definition of eCohort Studies in Health Research: Narrative Literature Review
Source: JMIR Public Health Surveill. 2021 Jan 21;7(1):e24588. doi: 10.2196/24588 (PMC7861999; doi:10.2196/24588)
Supplement: Multimedia Appendix 1 [file publichealth_v7i1e24588_app1.docx]

Appendix 1: Coding

| **MAIN CODES** | **SUBCODES** |
| --- | --- |
| **Research question** |  |
| **Sampling** |  |
|  | Sampling method |
|  | Sample definition |
| **Recruitment** |  |
|  | Advertisement |
|  | Recruitment process |
|  | Recruitment setting |
|  | Consent |
| **Baseline data collection** |  |
|  | Baseline data collection tools |
|  | Baseline data sources |
| **Follow-up** |  |
|  | Time (frequency) |
|  | Follow-up data collection tools |
|  | Follow-up data sources |
|  | Attrition and other biases |
|  | Participant communication and outreach |
| **Analysis** |  |
|  | Data quality and completeness |
|  | External validity/ other biases |
|  | Data management |
| **Dissemination** |  |
